# Cognitive complaints in age-related chronic conditions: A systematic review

**DOI:** 10.1371/journal.pone.0253795

**Published:** 2021-07-07

**Authors:** Nikki L. Hill, Sakshi Bhargava, Monique J. Brown, Hyejin Kim, Iris Bhang, Kaitlyn Mullin, Kathleen Phillips, Jacqueline Mogle

**Affiliations:** 1 College of Nursing, Pennsylvania State University, University Park, Pennsylvania, United States of America; 2 Department of Epidemiology and Biostatistics, Arnold School of Public Health, University of South Carolina, Columbia, South Caroline, United States of America; 3 South Carolina SmartState Center for Healthcare Quality, Arnold School of Public Health, University of South Carolina, Columbia, South Carolina, United States of America; 4 Rural and Minority Health Research Center, Arnold School of Public Health, University of South Carolina, Columbia, South Carolina, United States of America; 5 Office of the Study on Aging, Arnold School of Public Health, University of South Carolina, Columbia, South Caroline, United States of America; 6 Department of Biobehavioral Nursing and Health Informatics, School of Nursing, University of Washington, Seattle, Washington, United States of America; 7 Eberly College of Science, Pennsylvania State University, University Park, Pennsylvania, United States of America; 8 Penn State University Libraries, Pennsylvania State University, University Park, Pennsylvania, United States of America; 9 Edna Bennett Pierce Prevention Research Center, Pennsylvania State University, University Park, Pennsylvania, United States of America; Cardiff University, UNITED KINGDOM

## Abstract

**Introduction:**

Cognitive complaints in older adults may be indicative of progressive cognitive decline including Alzheimer’s disease (AD), but also occur in other age-related chronic conditions, complicating identification of early AD symptoms. To better understand cognitive complaints in aging, we systematically reviewed the evidence to determine their prevalence and characterization among older adults with the most common age-related chronic conditions.

**Methods:**

This systematic review was conducted in accordance with the Preferred Reporting Items for Systematic Reviews and Meta-Analyses statement and the review protocol was prospectively registered with PROSPERO (ID: CRD42020153147). Searches were conducted in PubMed, CINAHL, PsycINFO, Web of Science, and ProQuest Dissertations & Theses A&I in June 2020. Two members of the review team independently determined article eligibility for inclusion and conducted quality appraisal. A narrative synthesis of results was used to integrate findings across studies and draw conclusions regarding the strength of the evidence in each chronic condition category.

**Results:**

Thirty-seven articles met eligibility criteria and were included in the review. Conditions represented were diabetes (n = 20), heart disease (n = 13), hypertension (n = 10), chronic lung disease (n = 5), arthritis (n = 4), heart failure (n = 2), and hyperlipidemia (n = 2). In addition, 16 studies included a measure of multimorbidity. Overall, there was a higher prevalence of cognitive complaints in individuals with higher multimorbidity, including a potential dose-dependent relationship. Findings for specific conditions were inconsistent, but there is evidence to suggest that cross-sectionally, older adults with diabetes, heart disease, chronic lung disease, and arthritis have more cognitive complaints than those without these conditions.

**Conclusion:**

There is strong evidence demonstrating that cognitive complaints are more common in older adults with higher multimorbidity, but little research examining these associations over time. Improving our understanding of the longitudinal trajectory of cognitive complaints, multimorbidity, and objective cognition in older age is an important area for future research.

## Introduction

Cognitive complaints (i.e., self-reported problems with memory or other aspects of cognition) are often the first indicator of cognitive decline or Alzheimer’s disease (AD) in older adults [1, 2]. However, these symptoms can also be due to changes in physical health. As many as 60% of older adults with a chronic physical health condition (hereafter, chronic condition) report some type of cognitive complaint [3], compared to about 25% of older adults overall [4, 5]. Furthermore, many of the most common chronic conditions in older adults are thought to contribute to cognitive decline, and their associated symptoms may mimic the earliest signs of AD [6]. Characterizing trends in cognitive complaints within and across common chronic conditions is an important step in distinguishing symptoms that are indicative of progressive cognitive decline.

There is increasing evidence that the mechanisms of cognitive aging are largely shared with age-related chronic disease [e.g., inflammation, cellular damage; 7]. This is particularly notable in conditions that compromise cardiovascular health, including those that are among the most common in older adults such as hypertension, hyperlipidemia, coronary artery disease, and diabetes [8, 9]. For example, hypertension and diabetes convey a higher risk for late-life cognitive decline, mild cognitive impairment (MCI), and AD [10–13], and comorbidity of these conditions is known to compound cognitive decline risk. There is also evidence to suggest that older adults with higher multimorbidity (i.e., two or more chronic conditions) are more worried about their perceived forgetfulness [14]. This is an important consideration since cognitive complaints accompanied by worry are associated with a two-fold [15] to four-fold [16] higher risk of developing AD compared to complaints alone.

Given associations among cognitive complaints, comorbidity, and future AD risk, we systematically reviewed the evidence regarding cognitive complaints in the chronic conditions most common in older adults, excluding individuals with dementia. We focused on older adults specifically due to the differences in reporting of cognitive complaints across middle- and older age [17, 18] as well as the implication of cognitive complaints as an early indicator of non-normative cognitive decline. We sought to answer the following questions:

1. What is the prevalence of cognitive complaints in older adults with the most common are-related chronic conditions (i.e., hypertension, hyperlipidemia, arthritis, heart disease, diabetes, kidney disease, heart failure, chronic lung disease, or multimorbidity)?
2. How are these complaints characterized within and across conditions (e.g., type or severity of complaint, associations with specific conditions, impact on health or function, etc.)?

## Methods

We conducted a systematic review in accordance with the Preferred Reporting Items for Systematic Reviews and Meta-Analyses (PRISMA; [19]) statement. The review protocol was registered with PROSPERO (ID: CRD42020153147; [20]) prior to beginning the literature search. Due to the heterogeneity of studies identified, including differences in chronic conditions investigated and study designs, findings were described and integrated using narrative synthesis [21]. Covidence systematic review software was used to manage the search results and selection process [22].

### Search strategy

Searches were conducted on June 30, 2020 in the following databases: PubMed (Medline), CINAHL, PsycINFO, Web of Science, and ProQuest Dissertations & Theses A&I. Reference lists of identified articles were also inspected for other relevant studies. Search terms were built based on the primary concepts of ‘cognitive problems’ and ‘chronic conditions.’ In addition to searching for studies that examined chronic conditions generally or multimorbidity, searches were also conducted for the chronic conditions most common among U.S. older adults [23]: hypertension, hyperlipidemia, arthritis, heart disease (i.e., ischemic heart disease, coronary artery disease, or cardiovascular disease), diabetes, chronic kidney disease, heart failure, and chronic lung disease (i.e., COPD, chronic asthma, or chronic bronchitis). Keywords were generated for each primary concept by reviewing other related review articles and synonyms. The keywords were then combined with MeSH terms for PubMed (Medline) and Subject Headings (CINAHL). Search syntax for a sample database is presented in S1 Table. A grey literature search was conducted in conjunction with the formal database searches. Relevant government and professional association sites and Google Scholar were reviewed for additional conference proceedings, scholarly publications, and white papers.

The search was restricted to studies that were published in English and included adults aged 65 years or older, with no date limits. Dissertations and Theses did not have an option to limit based on age; therefore, search results from this database included all age ranges. Studies that identified the keywords in the title, abstract, or MeSH heading were retained, and all results were imported into Covidence for review.

### Selection criteria

Selected studies met the following inclusion criteria: 1) measure of one of the chronic conditions of interest and/or a measure of multimorbidity; and 2) measure of self-reported cognitive complaints. In addition, articles were excluded for the following: 1) sample mean age of less than 50 years or lifespan samples for which results were not available for an older adult subgroup; 2) included only individuals with dementia or results were combined for those with and without dementia; 3) setting in acute care or skilled nursing; 4) study protocols, editorials, reviews, books and book chapters, or interventional studies; and 5) studies of conditions that were not part of our review focus such as stroke, traumatic brain injury, Parkinson’s disease, cancer, sleep disorders, schizophrenia, or other psychiatric disorders. In addition, we excluded qualitative studies following our selection process since the number identified (n = 2) precluded meta-synthesis.

### Selection and extraction

Covidence was used to manage study selection and evaluation. During the abstract and title review stages, articles were independently reviewed by two authors to determine eligibility, and any disagreement was adjudicated by the first author. Data extracted from each article were entered into an evidence table and verified by a second author.

### Critical appraisal

We assessed risk of bias using a modified version of the Critical Appraisal Skills Programme (CASP; [24]) Checklists. All studies were evaluated for five core criteria: sampling frame, measurement of exposure (chronic conditions), measurement of outcome (cognitive complaints), treatment of confounding factors, and adequacy of analysis. Longitudinal studies were evaluated for two additional criteria: adequacy of follow-up period and approach to attrition. Each article was assessed by two authors independently, and any disagreements were adjudicated by the first author.

### Data synthesis

A narrative synthesis of results was used to integrate findings across studies, based on guidance by Popay et al. [25] to enhance the comprehensiveness and transparency of our methods. First, we reviewed and organized study results based on chronic condition type. Next, we examined cognitive complaint prevalence rates or trends as well as the direction and strength of effects, as available, to determine patterns across studies. Third, we categorized studies based on design (cross-sectional or longitudinal) and sample characteristics (e.g., size and setting), then explored relationships within and across categories, including differences and similarities. Finally, we drew conclusions regarding the strength of the evidence for each chronic condition category based on synthesis of results and quality appraisal.

## Results

**Fig 1:**
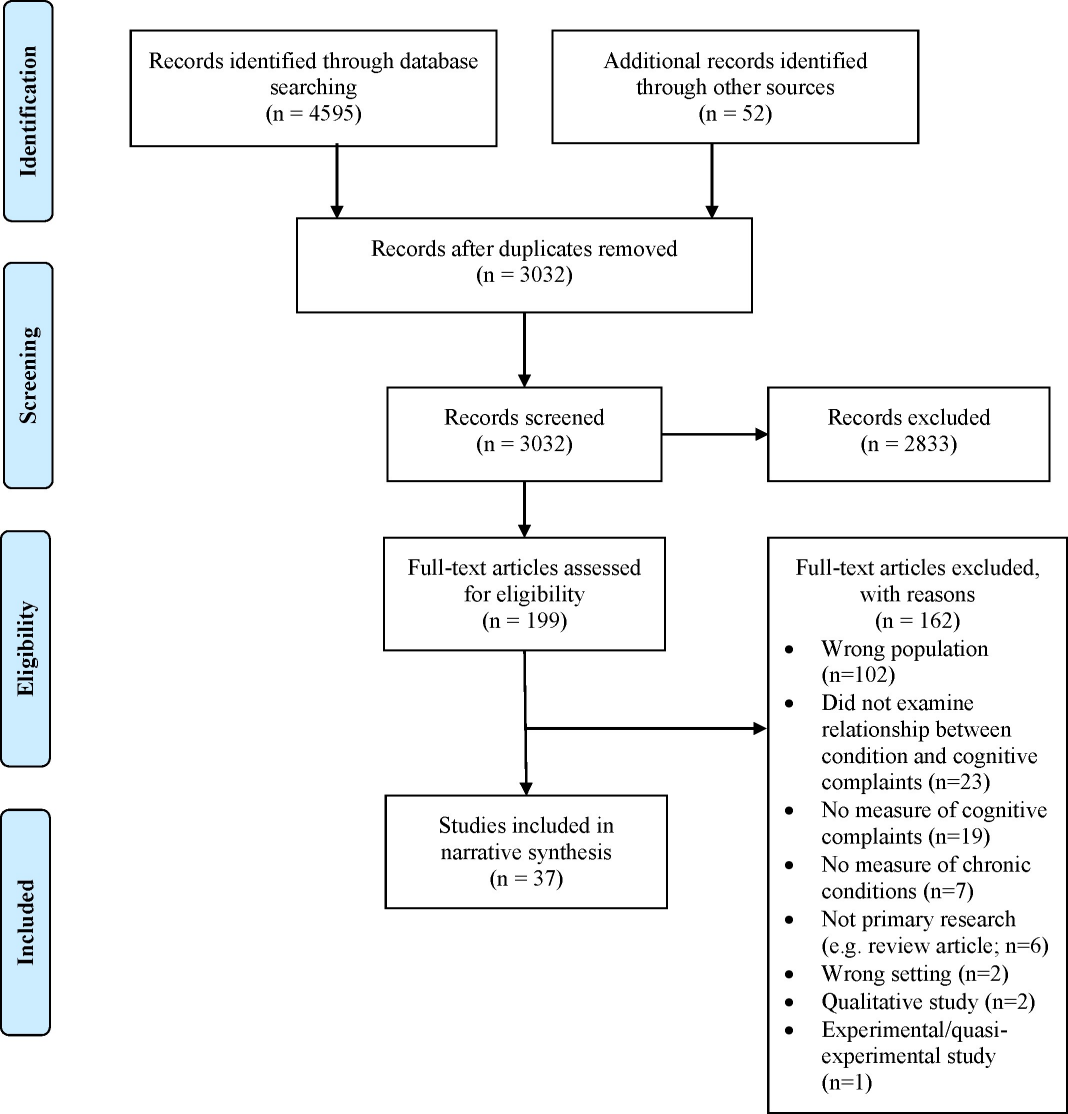
PRISMA flow diagram.

A total of 3,032 articles were identified from database and hand searches after duplicates were removed (see Fig 1 for details). After title and abstract review, 199 full-text articles were examined and ultimately, 37 studies met inclusion and exclusion criteria. Study details are provided in S2 Table.

### Study characteristics

Most (n = 31) studies were cross-sectional or only included cross-sectional results that were applicable to the purpose of our review; six longitudinal studies were identified. Sample sizes ranged from 66 to 227,393, and participants were recruited from a variety of settings: community-based (23 studies), primary care (10 studies), memory clinics (2 studies), and a university setting (1 study). Countries represented were Australia, Canada, China, England, Japan, Malaysia, The Netherlands, Norway, Singapore, South Korea, Spain, Sweden, United States, as well as three studies conducted across multiple countries. Although we excluded studies and results specific to individuals with dementia from our review, four studies included individuals with MCI in their samples along with participants who were cognitively normal.

Most studies examined cognitive complaints in specific conditions: diabetes (n = 20), heart disease (n = 13), hypertension (n = 10), chronic lung disease (n = 5), arthritis (n = 4), heart failure (n = 2), and hyperlipidemia (n = 2). In addition, 16 studies included a measure of multimorbidity. Cognitive complaint measures varied extensively across studies and all measured memory (n = 37). However, other cognitive domains were also represented, including perceptual-motor function (n = 7), attention (n = 5), executive function (n = 5), language (n = 4), and orientation (n = 2). Twenty-one studies used a multi-item measure of cognitive complaints. These included twelve validated measures, although there was little overlap: the Cognitive Failures Questionnaire (CFQ) was used in three studies, the Cognitive Difficulties Scale (CDS) in two studies, and all others were used in a single study. Investigator-developed multi-item measures were comprised of two-to-four questions. Sixteen studies used a single item to measure cognitive complaints, and all of these asked about memory specifically (e.g., “Do you have problems with your memory?”).

### Critical appraisal summary

Across studies, five met all applicable CASP quality appraisal criteria, and the remaining 32 varied in the type and number of criteria met. The most common potential sources of bias were in the measurement of chronic conditions or cognitive complaints, which were often assessed by self-report or a single question, respectively. Details of the critical appraisals for all studies are presented in Table 1.

**Table 1:** Quality appraisal by study using modified CASP criteria.

	All Studies					Longitudinal Studies	
Study	Was the sample adequate for examining the research question?	Was the exposure (chronic conditions) accurately measured to minimize bias?	Was the outcome (cognitive complaints) accurately measured to minimize bias?	Were confounding factors identified and appropriately addressed in the design and/or analysis?	Are the analyses adequate for answering the research questions(s)?	Was the follow-up period with participants long enough to answer the research question(s)?	Were strategies to address attrition sufficient?
Aarts et al. (2010)	✓	X	X	✓	✓		
Almkvist et al. (2017)	✓	X	✓	X	✓		
Argyropoulou et al. (2019)	X	X	✓	X	X		
Asimakopoulou et al. (2002)	✓	X	✓	X	✓		
Bassett et al. (1993)	✓	?	X	✓	✓		
Begum et al. (2012)	✓	X	✓	✓	✓		
Benito- León et al. (2010)	✓	?	X	✓	✓		
Bruce et al. (2019)	✓	✓	X	✓	✓		
Brunette et al. (2018)	✓	✓	✓	X	✓		
Caracciolo et al. (2013)	✓	✓	X	✓	✓		
Chen et al. (2014)	✓	X	X	X	✓		
Coomjis et al. (2002)	✓	X	X	✓	✓	✓	✓
Fischer et al. (2010)	X	?	✓	✓	✓		
Gunstad et al. (2006)	X	✓	✓	X	X		
Hao et al. (2019)	✓	X	X	X	X		
Harwood et al. (2004)	✓	X	✓	✓	✓		
Jorm et al. (2004)	✓	X	X	✓	✓		
Kuiper et al. (2017)	✓	X	X	✓	✓	X	✓
Kyrscio et al. (2014)	✓	?	?	?	✓	✓	✓
Lee et al. (2020)	✓	✓	✓	✓	✓		
Lourenco et al. (2018)	✓	X	X	✓	✓		
Matsuzawa et al. (2012)	✓	✓	✓	✓	✓		
Mewton et al. (2014)	✓	X	X	X	✓		
Nguyen et al. (2016)	✓	✓	✓	✓	✓		
Pedro et al. (2016)	✓	X	X	X	✓		
Peters et al. (2019)	✓	✓	X	?	✓	✓	?
Selnes et al. (2019)	✓	✓	X	✓	✓	✓	?
Shamshirgaran et al. (2019)	✓	X	X	✓	✓		
Taylor et al. (2018)	✓	X	X	X	✓		
Taylor et al. (2020)	✓	X	X	X	✓		
Tun et al. (1987)	✓	✓	✓	✓	✓		
Uiterwijk et al. (2014)	✓	✓	✓	✓	✓		
van den Kommer et al (2014)	✓	X	X	✓	✓	✓	✓
Wessels et al. (2007)	✓	?	✓	✓	✓		
Wong et al. (2012)	✓	✓	X	✓	✓		
Yap et al. (2020)	✓	X	✓	✓	✓		
Zhang et al. (2017)	?	X	X	✓	✓		

*Note*. ✓ = Yes; X = No;? = Unable to determine based on information provided. Shaded cells indicate studies were cross-sectional. CASP = Critical Appraisal Skills Programme.

### Cognitive complaint prevalence and characterization

In the following sections, results are synthesized based on type of chronic condition examined. Within each section, evidence regarding cognitive complaint prevalence is summarized first, followed by characterization (e.g., association with specific conditions, impact on function, etc.) of the complaints.

#### Multimorbidity and cognitive complaints

*Prevalence of cognitive complaints and multimorbidity*. Across the 16 studies that measured multimorbidity, the majority found that cognitive complaints among older adults were more common in those with more chronic conditions, and that complaints increased with multimorbidity burden. In a large (n = 220,211) U.S. population-based sample, Taylor et al. [26] found that among adults 65 and over, cognitive complaints were reported in 10% of those who reported one condition, 13% of those who reported two, and 21% of those who reported three or more. The prevalence of cognitive complaints in individuals with one self-reported chronic condition was 1.6 times more than in those who reported none, 1.5 times more for individuals who reported two compared to those with one or none, and 2.1 times more for individuals who reported three or more conditions than for two or less. A population-based Spanish study of older adults (n = 1,342) found similar results such that the percent of older adults reporting cognitive complaints increased with the number of chronic conditions reported: 21% in those with none, 23% in those with one, 26% in those with two or three, 34% in those with four or more [27]. In a sample drawn from primary care, Begum and colleagues [28] found cognitive complaints in 16% of those who reported three or fewer conditions, 32% of those reporting four or five, and 53% of those reporting six; in those who reported seven or more conditions, the prevalence dropped to 31%.

Two studies examined comorbid chronic conditions in those with and without cognitive complaints. Taylor and colleagues [26] found that the prevalence of reporting at least one chronic condition was higher in those with cognitive complaints (86%) compared to without (74%). And in a longitudinal study from the Netherlands (n = 2,032), participants with cognitive complaints reported more chronic conditions than those without complaints over a six-year period [29]. However, two studies found no differences in multimorbidity in those with and without cognitive complaints: one with exclusively male participants from the U.S., Canada, and Puerto Rico (n = 7,540) [30], and the other in a memory clinic sample in China (n = 53) [31].

*Characterization of cognitive complaints and multimorbidity*. Cross-sectionally, the strongest evidence supports a relationship such that higher multimorbidity is associated with the presence or severity of cognitive complaints [3, 14, 27, 28, 32–34]. Higher multimorbidity was associated with memory complaints [32, 34], poorer memory rating [33], and higher likelihood of reporting memory problems [27]. These associations persisted in studies that adjusted for multiple potential confounding factors such as age, gender, education, objective cognition, and psychological stress [14, 28]. Caracciolo et al. [3] also reported a dose dependent relationship such that the odds of cognitive complaints increased with number of chronic conditions. Taylor, Bouldin, and McGuire [35] found that in those with perceived cognitive decline, the prevalence of a related functional limitation was higher (40%) among those who reported at least one condition compared to those who reported none (25%). Aarts et al. [14] examined differences in associations based on the type of cognitive complaint. Multimorbidity was associated with forgetfulness, with a stronger relationship in those 55–69 years compared to those 70 and older. However, perceived memory decline over the past year was not associated with multimorbidity. There were four studies that found no association between multimorbidity and cognitive complaints [36–39].

Two studies examined longitudinal relationships and report mixed associations over time. Coomjis et al. [29] followed participants over a period of six years and found that changes in memory complaints across the three measurement occasions were associated with more chronic conditions. However, van den Kommer [40] found that the number of chronic conditions at baseline was not related to incident memory complaints after three years.

#### Diabetes

*Prevalence of cognitive complaints and diabetes*. Diabetes was the most studied chronic condition in our review, with 20 studies. The prevalence of cognitive complaints varied widely and was not always compared for differences in those with and without diabetes. In the largest study (total N = 220,221; [26]), 15% of those who reported diabetes had cognitive complaints compared to 11% of those who did not (*p* < .001). In another large multiethnic study of individuals who reported having diabetes (n = 23,112), 24% had cognitive complaints [41]. Matsuzawa et al. [42] examined prevalence among outpatient clinic patients of the University Hospital in Japan (n = 441) and found that in those with diabetes and no cognitive impairment (n = 261), 70% had memory complaints, 30% said other people found them forgetful, and 82% said they often used notes to avoid forgetting. This study also examined cognitive complaints in persons without diabetes; however, differences between groups were not tested. Yap et al. [34] found a small difference in cognitive complaint prevalence between a group characterized by both self-reported diabetes and hypertension (65%) compared to a low comorbidity group (59%). Studies that directly compared prevalence between older adults with and without self-reported diabetes tended to find no significant differences regardless of the type of complaint, including word finding difficulty, problems remembering new information, and perceived memory decline [30, 31, 43, 44].

*Characterization of cognitive complaints and diabetes*. The evidence regarding associations between diabetes and cognitive complaints is mixed. Some studies found that older adults who reported having diabetes, compared to those who did not, also reported more frequent or severe memory problems [36, 45, 46], poorer memory rating [33], and had greater odds of reporting memory problems [47]. However, other studies found no greater risk for cognitive complaints [48, 49], and no differences in composite complaint scores [50–52]. Wong et al. [44] found no difference in progressive forgetfulness in those with and without self-reported diabetes, and two studies found negative associations such that reporting diabetes was associated with fewer cognitive complaints [3, 53]. In the one longitudinal study identified, Kyrscio et al. [54] found that self-reported diabetes predicted a decreased likelihood of future transition from no complaint to having memory complaints.

#### Heart disease

*Prevalence of cognitive complaints and heart disease*. Heart disease, including coronary artery and cardiovascular disease, was examined in 12 studies, but few reported the prevalence of cognitive complaints. In a large (n = 220,221) sample representative of the U.S., adults 65 or older who reported a history of heart disease had significantly higher prevalence of cognitive complaints (18.3%) compared to those without (10.2%) [26]. Two studies reported the prevalence of cognitive complaints among coronary artery bypass grafting (CABG) patients. In a longitudinal study, all participants (n = 232) had coronary artery disease and those undergoing CABG had a higher prevalence of memory complaints at 12 months (39%) compared to controls not having surgery (14%) [55]. In another study with exclusively male participants (n = 7,540), history of CABG did not differ between those with and without cognitive complaints [30]. Similarly, Hao et al. [31] compared memory clinic patients with and without cognitive complaints and found no difference in reports of cardiovascular or heart disease.

*Characterization of cognitive complaints and heart disease*. Relationships between heart disease and cognitive complaints were inconsistent across studies, with some finding a positive association [33, 34, 49, 55] and others no link [40, 48, 51, 56]. In a large sample (n = 33,126) representing 16 European countries, reporting a history of heart attack was associated with poorer memory rating [33]. Similarly, in an Australian sample (n = 2,546), Jorm et al. [49] found that those with memory complaints and those who had seen a doctor about their memory were more likely to report “heart troubles.” In a sample from rural Malaysia (n = 6,179), cognitive complaints were associated with self-reported heart disease, and those with complaints had the highest odds of having reporting heart disease (followed by stroke, arthritis, and asthma; [34]). Gunstad et al. [57] found that while, on average, older adults with cardiovascular disease reported rarely or occasionally experiencing cognitive difficulties (*M* = 37.3; SD = 21.6) on the Cognitive Difficulties Scale (total score range: 0–152; [58]), item analysis showed the most difficulty with self-reported sustained attention and language abilities. In the Selnes et al. [55] study, self-reported changes in memory at three months were significantly higher among CABG patients, and the relative risk of developing new self-reported memory symptoms between three and 12 months was 2.5 times higher among CABG than non-CABG coronary artery disease patients.

Conversely, several studies did not support a relationship between heart disease and cognitive complaints. Benito- León et al. [48] compared Spanish older adults with cognitive complaints to a matched control group without complaints (n = 2,146) and found that those who reported heart disease were not more likely to report memory problems. Similarly, self-reported heart disease did not predict complaints in a memory clinic sample [51]. In addition, the two longitudinal studies that examined these relationships found no link: older adults who reported cardiovascular disease were not more likely to report incident memory complaints after three years [40], and neither arrythmia nor myocardial infarction were associated with incident complaints after 1.5 years [56]. However, in the Kuiper et al. [56] study, reporting cardiovascular disease decreased the odds of no longer having cognitive complaints at follow-up.

#### Hypertension

*Prevalence of cognitive complaints and hypertension*. Across the 10 studies that examined hypertension, evidence is inconsistent regarding cognitive complaint prevalence. In a study of older adults with hypertension recruited from 13 countries (n = 2,295), 36.4% reported cognitive complaints [59]. The study by Yap et al. [34] (previously discussed above for diabetes) found a difference in complaint prevalence between a group characterized primarily by self-reported comorbid hypertension and diabetes (65%) compared to a low comorbidity group (59%), but this was not significant when examining hypertension alone. Three studies examined the prevalence of reported hypertension among those with and without cognitive complaints and found no differences in community-based [30, 60] and memory clinic [31] samples.

*Characterization of cognitive complaints and hypertension*. Studies with larger samples found associations between hypertension and cognitive complaints, including higher odds of self-reported memory impairment (nationally representative sample in U.S., n = 7,824; [47]) and poorer memory ratings (n = 33,126, 16 European countries; [33]). However, others found no cross-sectional or longitudinal relationship [34, 51, 59]. Among individuals recruited from a hypertension clinic in the Netherlands, the mean Cognitive Failures Questionnaire score was 33.7 out a possible 100 points, suggesting that individuals in the study reported experiencing cognitive problems rarely to occasionally [52]. In addition, two studies in older adults with hypertension found no difference in sitting systolic or diastolic blood pressure [59], duration of hypertension, or anti-hypertensive medications [60] between those with and without memory complaints.

#### Chronic lung disease

*Prevalence of cognitive complaints and chronic lung disease*. The most common chronic lung disease represented was COPD, but two studies examined chronic asthma or bronchitis. Only the study by Taylor and colleagues [26] reported prevalence, and they found that in adults 65 or older, cognitive complaints were reported in 21% of those with self-reported COPD compared to 10% of those without.

*Characterization of cognitive complaints and chronic lung disease*. Other studies had mixed results with two finding an association between chronic lung disorders and cognitive complaints [45, 49] and two not [48, 61]. In a large study (n = 18,633) conducted in Norway, reporting COPD was associated with poorer self-reported memory [45]. Jorm et al. [49] found that older Australians with memory complaints were more likely to report chronic asthma or bronchitis than those without complaints. However, Benito- León et al. [48] found that older adults with self-reported COPD were not at greater risk for cognitive complaints compared to those without. A smaller study (n = 95) focused on former smokers exclusively and found no differences in overall cognitive complaints between those with and without COPD [61].

#### Arthritis

*Prevalence of cognitive complaints and arthritis*. Four studies examined cognitive complaints in self-reported arthritis, two of which reported prevalence rates. Taylor and colleagues [26] found that cognitive complaints were more prevalent in U.S. older adults who reported arthritis (14.7%) compared to those who did not (8.6%). Prevalence rates were much higher among Malaysian older adults in the study by Yap et al. [34] although participants were compared by categories based on latent class analysis. In the arthritis group, characterized by a high conditional probability of arthritis and generally higher probabilities of all health conditions except diabetes, 79% reported cognitive complaints compared to 59% in the low comorbidity group and 65% in the diabetes and hypertension group.

*Characterization of cognitive complaints and arthritis*. Yap et al. [34] also found that older adults who reported arthritis had twice the odds of cognitive complaints compared to those in the low comorbidity group. Furthermore, Jorm et al. [49] found that older adults with cognitive complaints, as well as those who had seen a doctor about their memory, were more likely to report having arthritis. However, Almkvist et al. [45] found no association between arthritis and self-reported memory.

#### Heart failure

Two studies examined cognitive complaints in older adults who reported having heart failure, although neither provided prevalence rates. Reporting heart failure was associated with poorer self-reported memory in a cross-sectional investigation, including poorer self-reported short-term compared to long-term memory [45]. However, in a longitudinal study, self-reported heart failure at baseline was not associated with incident memory complaints at the 1.5 year follow-up [56].

#### Hyperlipidemia

Two studies examined cognitive complaints in hyperlipidemia, and both found no relationship. Hao et al. [31] found no difference in the prevalence of self-reported hyperlipidemia between those with and without cognitive complaints. A study by Uiterwijk et al. [52] included only older adults with hypertension, and in this sample, those with and without hypercholesterolemia had no difference in cognitive complaints.

## Discussion

In this systematic review, we examined the prevalence and characterization of cognitive complaints in the chronic physical health conditions that are most common among older adults (excluding dementia). We found strong support for a higher prevalence of cognitive complaints in those with higher multimorbidity, including an increase in complaints with more chronic conditions. There is some evidence that older adults with diabetes, heart disease, chronic lung disease, or arthritis have more cognitive complaints than those without these conditions, but given the extensive differences in measurement, direct comparisons cannot be made with other population estimates. In terms of characterization of cognitive complaints, measurement differed substantially across studies; therefore, our conclusions are largely discussed in terms of specific conditions or multimorbidity.

One of the most consistent findings across studies was a link between multimorbidity and cognitive complaints: older adults with more chronic conditions tend to report more cognitive problems, and reciprocally, older adults with cognitive complaints tend to have more chronic conditions. Multimorbidity can include many different conditions, and indeed, the studies in our review used a variety of measures. Despite this heterogeneity, 12 of the 16 studies found a positive association between the two. Furthermore, in the four studies that reported no association, methodological factors may have contributed including small sample sizes (n = 67–182) [36–38], and a dichotomized multimorbidity variable [39].

Multimorbidity is associated with a higher risk for developing MCI, as well as an accelerated trajectory of cognitive decline [62], but our review suggests that the relationship between cognitive complaints and multimorbidity is not due exclusively to objective deficits in cognitive performance as most studies only included older adults without any evidence of cognitive impairment. Given that age-related chronic conditions may be linked with cognitive decline and AD risk, particularly those that impair cardiovascular health (e.g., hypertension), identifying older adults with multimorbidity who are experiencing early cognitive symptoms would support targeting preventive interventions to a high-risk group. Subjective cognitive decline (i.e., self-observed decline in cognitive function; SCD) has been proposed as an intermediary stage between normal cognition and MCI or AD [63, 64]; therefore, understanding cognitive complaints in the context of multimorbidity may help refine our understanding of the link between SCD and AD. Furthermore, medications commonly used to treat many chronic conditions can have cognitive side effects, particularly in older adults [65, 66]. The prevalence of taking multiple medications has dramatically increased in the past two decades: an over 50% increase in older adults taking two, and an almost 300% increase in those taking three or more [67]. Future research on SCD, multimorbidity, and AD risk should consider the potential role of medications and polypharmacy on cognitive outcomes.

Diabetes was the most frequently studied condition in our review, in line with its prevalence in aging. Almost 30% of older adults are diagnosed with diabetes in the U.S. [68] and it has been associated with cognitive deficits that progress to dementia in some individuals [69, 70]. Mixed findings across studies may be at least partially attributed to methodological heterogeneity, particularly in the measurement of cognitive complaints. For example, Bruce and colleagues [43] used a single-item question and did not find a significant difference in cognitive complaints between older adults with and without diabetes. However, Tun and colleagues [46] used a standardized questionnaire, the Short Inventory of Memory Experiences (SIME; [71]) and found that participants with diabetes reported more cognitive complaints than those without. Given the higher risk of dementia and MCI in persons with diabetes [72], further examination of cognitive complaints as a potential early indicator of this decline is needed to improve our understanding and interpretation of symptom trajectories.

We found mixed support for a link between cognitive complaints and heart disease, although the strongest evidence suggests that older adults with heart disease have more cognitive complaints than their peers. This is perhaps not surprising given known associations between cardiovascular and brain health [73, 74], as well as the scope of health conditions that either contribute to heart disease (e.g., atherosclerosis) or are included under its umbrella (e.g., coronary artery disease, arrhythmias, cardiomyopathy). Interestingly, we did not find strong evidence to support an association between cognitive complaints and hypertension (discussed below), although persistent hypertension can lead to hypertensive heart disease. It is important to note that to be as comprehensive as possible, we included multiple exposures in our heart disease category: coronary artery disease, cardiovascular disease, as well as a history of myocardial infarction and CABG. Although those who have undergone CABG, and almost all of those who have experienced a myocardial infarction, also have heart disease, the severity of their condition may be inherently different than other older adults with chronic heart disease and no acute events. There is little longitudinal research examining how the progression of heart disease may impact cognitive complaints; in the two studies identified, it did not predict greater future complaints. It may be that the health complications of heart disease take precedence over more subtle symptoms such as cognitive changes, but this is an area that requires further investigation.

Hypertension was not consistently related to cognitive complaints in our review. Among patients with hypertension, the prevalence of cognitive complaints was higher than that reported in the general population [35], but only the largest samples identified a significant relationship between self-reported hypertension and rates of cognitive complaints [33, 47]. Furthermore, eight of the ten studies that examined hypertension found no relationship. Critically, studies were also limited in their assessment approaches. Most used self-reported diagnosis of hypertension and a single item to characterize individuals as having a cognitive complaint, which restricts interpretation of our results. Despite the lack of clear evidence of a relationship between hypertension and cognitive complaints, hypertensive status is a known risk factor for objective cognitive impairment [75] and decline [76, 77], particularly at midlife [78]. It is possible that hypertension and cognitive complaints are too prevalent after age 65 to be able to identify a clear association. The biological processes underlying hypertension require additional time to impact brain functioning, and therefore cognitive performance [79]. Given that the studies in the current review did not account for age at diagnosis of hypertension, the mix of individuals with recent compared with long-term diagnoses could limit the likelihood of uncovering a relationship. Further, management of hypertension through interventions such as diet protects objective cognitive performance [80], and similar confounds were rarely considered in the studies in the current review [60].

The findings on the relationship between chronic lung disorders and cognitive complaints were inconsistent, showing both an association and a lack thereof. These mixed findings could be due to several reasons such as differences in design, sample, and operationalization of our measures of interest. For example, Taylor and colleagues [26] operationalized COPD through self-report, while Brunette et al. [61] used spirometry among former smokers. Nevertheless, the link between cognition and COPD seems to be a persistent relationship overall [81], and certain measures of cognitive complaints may be more sensitive to perceived changes. Future research should include assessing cognitive complaints and disease stage among COPD patients to determine if complaints increase with disease severity.

For the remaining chronic conditions represented in our review–arthritis, heart failure, and hyperlipidemia–there was limited evidence to draw conclusions about relationships. Arthritis did have relatively consistent links with cognitive complaints (three out of four studies). Inflammation is thought to play a role in cognitive aging [82], and low-grade systematic inflammatory processes may contribute to arthritis [83, 84]; however, some studies have found associations between arthritis and cognition [85, 86] while others have not [87]. For both heart failure and hyperlipidemia, there is not sufficient evidence to describe cognitive complaints specific to these conditions. It is important to note both conditions tend to be comorbid with heart disease–hyperlipidemia contributing to its development and heart failure often developing along with progressive coronary artery disease. Therefore, a more detailed examination of cognitive complaints in the multiple conditions linked to cardiovascular disease is needed to determine when complaints arise and how they change with disease severity.

There are several limitations to consider with this review. First, identified studies used a wide variety of cognitive complaint measures, ranging from single items to validated scales. Although there have been attempts to more precisely define cognitive complaints in aging, such as SCD [64], as well as recommendations for operationalization in research [88], existing evidence uses a variety of operational definitions, complicating synthesis across studies. Furthermore, only seven studies measured self-reported cognition in domains other than memory (e.g., executive function). This is an important limitation in current evidence as the type of cognitive complaint, including non-memory complaints, may help distinguish MCI and AD risk [89]. Second, while the studies included in this review cover a wide range of chronic conditions, we did not have sufficient representation of any one condition in some cases to draw conclusions. Also, our focus on the most common age-related chronic conditions meant that other conditions that are associated with cognitive decline risk, such as sleep disorders, were not included. Additionally, some studies that measured multimorbidity did not provide information about the chronic conditions included in their measure. Third, although several studies included clinical assessments to determine the presence of a condition, most relied on self-report. This approach is common, and often necessary in large studies, but does influence measurement validity, particularly when determining condition prevalence [90]. Future research should use clinical identification of conditions as well as a consistent operationalization of cognitive complaints, including measuring both severity as well as associated impact (e.g., on functional ability or well-being), to better understand complaints in the content of chronic conditions. Fourth, we planned to explore differences across sociodemographic categories, such as gender or race, but there was not consistent reporting of subgroup results across studies to support this approach. Given known health disparities in cognitive decline risk and multimorbidity among underrepresented groups, this is a critical area to address in future research. Finally, our review was limited to older adults without dementia; however, we did include samples comprised of individuals with normal cognition and MCI (four studies), to be as comprehensive as possible in our review. Although no thematic differences were identified in the results of these studies compared to others, self-report of both cognitive complaints and chronic conditions could be impacted by MCI. In addition, while the current review did identify six longitudinal studies, there is limited evidence to determine how cognitive symptoms may evolve and change over time.

### Conclusion

There is strong evidence demonstrating that cognitive complaints are more common in older adults with higher multimorbidity, but little research examining these associations over time. Given that the earliest symptom of AD is likely to be a report of memory or other cognitive problems and many chronic conditions increase the risk for AD, improving our understanding of the longitudinal trajectory of cognitive complaints, multimorbidity, and objective cognition in older age is an important area for future research. Disparate findings regarding cognitive complaints in specific age-related chronic conditions suggest that the heterogeneity in operationalization of key measures, as well as the common use of single-items and self-report, hinders our ability to draw definitive conclusions with current evidence. However, given that cognition is closely linked with other aspects of health, particularly cardiovascular function, cognitive complaints should be further investigated as an early indicator of cognitive decline in older adults with higher AD risk due to chronic conditions.

## Supporting information

S1 Checklist PRISMA checklist. (DOC)

S1 Table Search syntax for sample database. (DOCX)

S2 Table Study details and relevant results. (DOCX)
