# Supplementary material for: Cognitive complaints in age-related chronic conditions: A systematic review
Source: PLoS One. 2021 Jul 7;16(7):e0253795. doi: 10.1371/journal.pone.0253795 (PMC8263303; doi:10.1371/journal.pone.0253795)
Supplement: S1 Table — (DOCX) [file pone.0253795.s002.docx]

**S1 Table. Search Syntax for Sample Database**

| (((("subjective memory"[Title/Abstract]) OR ("subjective cognit*"[Title/Abstract]) OR ("cognitive self report"[Title/Abstract]) OR ("cognitive complaint*"[Title/Abstract]) OR ("cognitive concern*"[Title/Abstract]) OR ("Cognitive difficult*"[Title/Abstract]) OR ("Cognitive failure*"[Title/Abstract]) OR ("Cognitive frailty"[Title/Abstract]) OR ("Cognitive dysfunction"[Title/Abstract] OR "Cognitive Dysfunction"[Mesh]) OR ("Cognitive problem*"[Title/Abstract]) OR ("Memory self report"[Title/Abstract]) OR ("Memory complaint*"[Title/Abstract]) OR ("Memory concern*"[Title/Abstract]) OR ("Memory difficult*"[Title/Abstract]) OR ("Memory lapse*"[Title/Abstract]) OR ("Memory problem*"[Title/Abstract]) OR ("Self reported memory"[Title/Abstract]) OR ("Self reported cognit*"[Title/Abstract]) OR ("Functional memory"[Title/Abstract]) OR ("Functional cognit*"[Title/Abstract]) OR ("Forgetfulness"[Title/Abstract]) OR ("Meta-cognition"[Title/Abstract] OR "metacognition"[Title/Abstract] OR "Metacognition"[Mesh]) OR ("Meta-memory"[Title/Abstract] OR "metamemory"[Title/Abstract]) OR ("Everyday memory"[Title/Abstract]) OR ("Everyday cognit*"[Title/Abstract]) OR ("Memory perception*"[Title/Abstract]) OR ("Perceived memory"[Title/Abstract]) OR ("Difficulty concentrating"[Title/Abstract])) AND ((("chronic condition"[Title/Abstract]) OR ("chronic illness"[Title/Abstract]) OR ("chronic disease"[MeSH Terms]) OR ("chronic disease"[Title/Abstract]) OR ("morbidity"[MeSH Terms]) OR ("morbidity"[Title/Abstract]) OR ("comorbidity"[MeSH Terms]) OR ("comorbidity"[Title/Abstract]) OR ("multimorbidity"[MeSH Terms]) OR ("multimorbidity"[Title/Abstract]) OR ("chronic health condition"[Title/Abstract]) OR ("long-term illness"[Title/Abstract]) OR ("chronic disorder"[Title/Abstract]) OR ("Chronic medical condition"[Title/Abstract])) OR (("hypertension"[MeSH Terms]) OR ("hypertension"[Title/Abstract]) OR ("hypercholesterolemia"[MeSH Terms]) OR ("hypercholesterolemia"[Title/Abstract]) OR ("high cholesterol"[Title/Abstract]) OR ("arthritis"[MeSH Terms]) OR ("arthritis"[Title/Abstract]) OR ("coronary disease"[MeSH Terms]) OR ("coronary disease"[Title/Abstract]) OR ("coronary heart disease"[Title/Abstract]) OR ("diabetes mellitus"[MeSH Terms]) OR ("diabetes mellitus"[Title/Abstract]) OR ("diabetes"[Title/Abstract]) OR ("heart failure"[MeSH Terms]) OR ("heart failure"[Title/Abstract]) OR ("COPD" [MeSH Terms]) OR ("COPD" [Title/Abstract]) OR ("chronic obstructive pulmonary disease"[MeSH Terms]) OR ("chronic obstructive pulmonary disease"[Title/Abstract]) OR ("kidney disease"[MeSH Terms]) OR ("kidney disease"[Title/Abstract]))) |
| --- |
