# Supplementary material for: Cognitive complaints in age-related chronic conditions: A systematic review
Source: PLoS One. 2021 Jul 7;16(7):e0253795. doi: 10.1371/journal.pone.0253795 (PMC8263303; doi:10.1371/journal.pone.0253795)
Supplement: S2 Table — (DOCX) [file pone.0253795.s003.docx]

**S2 Table. Study Details and Relevant Results**

| **Study** | **Design** | **Country** | | **Age**  **M (SD)** | **Sample** | **Measures** | | **Results** |
| --- | --- | --- | --- | --- | --- | --- | --- | --- |
|  |  |  |  |  |  | **Chronic Conditions** | **Cognitive Complaints** |  |
| Aarts et al. (2010)^14^ | Cross-sectional | The Netherlands | | 70 (NR) | N = 15,188  Community-dwelling, representative of those 55+ in Limburg | Self-report: # of medical conditions | "Do you perceive yourself as forgetful?" If yes, rate the degree of worry and if worsened over the last year | Multimorbidity was associated with memory complaints - relationship stronger among those 55-69 than 70 and older  Worry about memory was only associated with 3+ conditions  Multimorbidity not associated with perceived memory decline |
| Almkvist et al. (2017)^45^ | Cross-sectional | Norway | | 66.8 (8.4) | N = 18,633  Population-based | Self-report: 10 disease types | Metamemory Questionnaire (MMQ) | Self-reported COPD, diabetes, heart failure were associated with memory complaints  Self-reported osteoarthritis, rheumatoid arthritis, asthma, kidney diseases, other heart diseases not associated with memory complaints |
| Argyropoulou et al. (2019)^36^ | Cross-sectional | Spain | | 68.8 (5.4) | N = 67  Continuing education students | Self-report: 19 mental and physical diseases | Memory Complaint Questionnaire (MAC-Q) | Memory complaints not associated with multimorbidity  Persons who reported diabetes reported more memory problems than their counterparts |
| Asimakopoulou et al. (2002)^50^ | Cross-sectional | England | | 62.4 (9.6) | N = 66  General practice, matched groups | Self-report supported by medical records: diabetes | Subjective Memory Questionnaire (SMQ) | No significant differences in SMQ scores between the group who reported having diabetes and the control group |
| Bassett et al. (1993)^32^ | Cross-sectional | United States | | NR (65+ years) | N = 228  Drawn from Eastern Baltimore Mental Health Survey | Self-report: # of current illnesses | "Do you find that you have trouble with your memory?" | Number of self-reported current illnesses predicted memory complaints |
| Begum et al. (2012)^28^ | Cross-sectional | England | | 74.6 (6.9) | N = 126  Primary care patients | Self-report: 14 physical health conditions | Geriatric Mental State interview | % memory complaints by # of self-reported medical conditions:   - 0-3 conditions: 16.3% - 4 conditions: 32% - 5 conditions: 33.3% - 6 conditions: 53.3% - 7+ conditions: 30.6%   Memory complaints were positively associated with # of conditions |
| Benito-León et al. (2010)^48^ | Cross-sectional | Spain | | 75.7 (5.8) | N = 2,146  Drawn from population-based study: Neurological Disorders in Central Spain | NR | “Do you suffer from forgetfulness since the last interview?” (Y/N) | Diabetes, heart disease, and COPD were not significant predictors of memory complaints |
| Bruce et al. (2019)^43^ | Cross-sectional | Australia | | 71.2 (8.8) | N = 340  Drawn from matched cohort study: Busselton Diabetes Study | Clinical assessment: diabetes | “Do you consider yourself to have difficulty with your memory?” “Do you have word-finding difficulty?” “Do you have memory difficulty for new names/dates?” “Has your memory become worse over time?” | No significant differences between those with and without diabetes on any self-reported memory questions  Diabetes was associated with fewer self-reported memory problems |
| Brunette et al. (2018)^61^ | Cross-sectional | United States | | 68.7 (7.0) | N = 95  Former smokers, drawn from COPDGene Study | Pulmonary function testing: COPD | Cognitive Difficulties Scale (CDS) | No difference in cognitive difficulties between COPD and no COPD groups |
| Caracciolo et al. (2013)^3^ | Cross-sectional | Sweden | | No complaints: 71.9 (3.2)  Complaints: 73.1 (5.9) | N = 11,379  Drawn from Swedish Twin Registry | Medical records, self-report, and informant report: 8 disease groups | Self-reported memory change within the last 3 years | Circulatory, musculoskeletal, respiratory, gastrointestinal, and urological diseases, but not endocrine, were significantly associated with memory complaints  Dose-dependent relationship between multimorbidity and odds of memory complaints |
| Chen et al. (2014)^47^ | Cross-sectional | United States | | 70.1 (6.3) | N = 7,824  Drawn from Gallup Poll lifespan study | Self-report: hypertension, diabetes | Single question about the presence of perceived memory problems | Self-reported hypertension and diabetes increased the odds of reporting memory problems |
| Comijs et al. (2002)^29^ | Longitudinal (6-year period) | Netherlands | No complaints: 68.3 (8.3)  Complaints: 69.8 (8.5) | | N = 2,032  Drawn from Longitudinal Aging Study Amsterdam | Self-report: # of chronic diseases | "Do you have complaints about your memory?" | At all 3 measurement occasions, participants with memory complaints reported more chronic diseases than those without complaints  Longitudinally, changes in memory complaints were associated with more chronic diseases |
| Fischer et al. (2010)^37^ | Cross-sectional | Canada | | 69.2 (10.0) | N = 85  Primary care patients | Cumulative Illness Rating Scale (CIRS) | Patient Assessment of Own Functioning (PAOF) | No correlation between medical co-morbidity and memory complaints |
| Gunstad et al. (2006)^57^ | Cross-sectional | United States | | 69.8 (7.9) | N = 84  Cardiology clinic patients | Medical history: CVD | Cognitive Difficulties Scale (CDS) | Average total CDS score of 37.3 (out of 100) for individuals who reported a history of CVD |
| Hao et al. (2019)^31^ | Cross-sectional | China | | NR (60+ years) | N = 135  Memory clinic patients | NR | SCD (plus) criteria | Chronic condition prevalence in normal cognition/SCD (plus):   - Hypertension 39%/40% - CVD 13%/13% - Heart disease 9%/7% - Diabetes 26%/23% - Hyperlipidemia 13%/37% - Multimorbidity 30%/30% |
| Harwood et al. (2004)^51^ | Cross-sectional | United States | | 74.1 (7.0) | N = 232  Memory clinic patients | Self-report: hypertension, heart disease, diabetes | Memory Questionnaire (MQ) | Self-reported diabetes, hypertension, and atherosclerotic heart disease did not predict MQ scores |
| Jorm et al. (2004)^49^ | Cross-sectional | Australia  Community-based | | No complaints:  62.5 (1.5)  Complaints:  62.4 (1.4) | N = 2,546  Drawn from PATH Through Life Project | Self-report: 10 chronic medical conditions | “Do you feel you remember things as well as you used to?“ “Does this memory problem interfere in any way with your day to day life?” “Have you seen a doctor about your memory?” | Self-reported diabetes: no differences across complaints/no complaints  Self-reported chronic bronchitis/asthma: more likely in complaints group  Those with memory complaints more likely to report heart troubles, arthritis, asthma/chronic bronchitis  Those who saw a doctor about memory more likely to report heart troubles, arthritis |
| Kryscio et al. (2014)^54^ | Longitudinal (up to 17+ years) | United States | | 73.2 (7.4) | N = 531  Drawn from Biologically Resilient Adults in Neurological Studies (BRAiNS) | Self-report: hypertension, diabetes | Asked if they had noticed any change in memory since their last visit | Self-reported diabetes (and not hypertension) predicted decreased likelihood of transition to having memory complaints |
| Kuiper et al. (2017)^56^ | Longitudinal (1.5 year period) | Netherlands | | 70 (4.4) | N = 8,762  Drawn from LifeLines Cohort Study | Self-report: cardiovascular conditions | "Do you have complaints about your memory?" | None of the three reported cardiovascular conditions (arrythmia, MI, heart failure) was associated with incident memory complaints  Self-reported arrythmia and MI were associated with SMC recovery |
| Lee et al. (2020)^38^ | Cross-sectional | Korea | | 78.4 (5.9) | N = 182  Community-dwelling, senior welfare center | Self-report: medical history | Cognitive Failure Questionnaire Korean version (CFQ-K) | Cognitive failures were positively associated with comorbidity |
| Lourenco et al. (2018)^33^ | Cross-sectional | 16 European countries | | 65.4 (10.0) | N = 33,126  Harmonized from Survey of Health, Ageing, and Retirement in Europe (SHARE) | Self-report: MI, diabetes, hyperglycemia, hypertension | “How would you rate your memory at the present time?” | Reports of more than two chronic diseases, diabetes or hyperglycemia, history of MI, and hypertension were associated with poorer memory rating |
| Matsuzawa et al. (2012)^42^ | Cross-sectional | Japan | | With diabetes: 72.7 (5.6)  Without diabetes: 72.6 (4.6) | N = 441  Hospital outpatient clinic | Medical chart review | Cambridge Examination for Mental Disorders for the Elderly | Comparison of with/without diabetes:   - Had memory complaints: 70.1%/67.2% - Others found them forgetful: 29.9%/30.4% - Use notes to avoid forgetting:  82.3%/86.4% |
| Mewton et al. (2014)^39^ | Cross-sectional | Australia | | NR (range = 65-85) | N = 1,905  Community-dwelling | Self-report: 21 chronic physical conditions | "Compared with others your age, how would you rate your memory?" "Compared with 5 years ago, how would you rate your memory?" | Report of one or more chronic conditions did not increase the risk for subjective memory impairment |
| Nguyen et al. (2016)^60^ | Cross-sectional | United States | | No complaints: 78.3 (5.5)  Complaints:  78.6 (4.9) | N = 105  Drawn from a longitudinal study on healthy cognitive aging | Self-report: hypertension | Asked participants to rate their overall problems with memory | No differences in prevalence of self-reported hypertension between those with and without memory complaints  Among individuals who reported hypertension, those with and without memory complaints did not differ in duration of hypertension or number of antihypertensive medications |
| Pedro et al. (2016)^27^ | Cross-sectional | Spain | | 74.25 (6.68) | N = 1,342  Random sample drawn from Madrid census | Self-report and current medications: # of chronic conditions | "Do you have memory problems?"  "Do you forget where you put things?"  "Do you forget the names of family and acquaintances?" | % of memory complaints by chronic conditions:   - No conditions: 20.8% - 1 condition: 22.9% - 2-3 conditions: 26.1% - 4+ conditions: 34.1% - Asthma: 5.7% - High cholesterol: 21.4% - Other heart diseases: 12.2% - Hypertension: 38.6% - MI/angina: 4.5% - Diabetes: 10.1% |
| Peters et al. (2019)^59^ | Longitudinal (mean follow-up = 2.3 years) | 13 countries | | No complaints: 83.5 (3.1)  Complaints: 83.4 (3.1) | N = 2,295  Drawn from Hypertension in the Very Elderly Trial (HYVET) | Clinical assessment: hypertension | “Do you feel you have more problems with memory than most?” | 36.4% of individuals with hypertension reported memory complaints  No difference in blood pressure between those with and without memory complaints |
| Selnes et al. (2004)^55^ | Longitudinal (12-month period) | United States | | CABG: 63.4 (9.6)  Controls: 65.8 (9.2) | N = 232  Cardiology patients | Cardiac catheterization: Coronary artery disease | Report of decline in memory since previous interview | Prevalence of memory complaints at 12 months was higher among CABG patients (39%) than controls (14%)  Relative risk of reporting new memory complaints between 3 and 12 months was 2.5 times higher among CABG patients than controls |
| Shamshirgaran et al. (2019)^41^ | Cross-sectional | Australia | | NR (45+ years) | N = 23,112  Drawn from 45 and Up Study, individuals with diabetes | Self-report: diabetes and # of medical conditions | "In general, how would you rate your memory?" | Prevalence of SMC among individuals who reported diabetes: 23.7%. |
| Taylor, et al. (2018)^35^ | Cross-sectional | United States | | NR (45+ years) | N = 227,393  Population-based | Self-report: # of chronic conditions | SCD: “During the past 12 months, have you experienced confusion or memory loss that is happening more often or is getting worse?” | % SCD by self-reported chronic conditions:   - Any chronic condition: 15.2% - No chronic conditions: 5.2% |
| Taylor et al. (2020)^26^ | Cross-sectional | United States | | NR (45+ years) | N = 220,221  Population-based | Self-report: asthma, MI/angina, heart disease, COPD, emphysema,  chronic bronchitis, arthritis, kidney disease, diabetes | SCD: “During the past 12 months, have you experienced confusion or memory loss that is happening more often or is getting worse?” | Persons who reported heart disease and COPD had higher prevalence of SCD than those who did not  Prevalence of reporting at least one chronic condition was higher in those with SCD than those without: 86.3% vs. 73.5%   Prevalence of SCD in those who reported vs. did not report chronic disease:   - Heart disease: 18.3% vs. 10.2% - Diabetes: 15.2% vs. 10.6% - Asthma: 16.7% vs. 11.2% - COPD: 20.6% vs. 10.4% - Arthritis: 14.7% vs. 8.6% - 1 condition: 10.0% vs. 6.3% - 2 comorbid conditions: 13.0% vs. 8.5% - 3+ comorbid conditions: 20.9% vs. 9.7% |
| Tun et al. (1987)^46^ | Cross-sectional | United States | | With diabetes: 68.9 (2.9)  Without diabetes: 68.2 (3.1) | N = 144  Hospital outpatients, matched groups | Clinical assessment: diabetes | Short inventory of memory experiences (SIME)  Memory Problem Inventory (MPI) | Diabetes group reported more frequent and more severe memory problems |
| Uiterwijk et al. (2014)^52^ | Cross-sectional | The Netherlands | | 56.1 (12.1) | N = 109  Hypertension clinic patients | Clinical assessment: hypertension | Cognitive Failure Questionnaire (CFQ) | Mean CFQ score in individuals with hypertension was 33.7 (out of 100)  Participants with and without hypercholesterolemia did not differ on subjective cognitive failures  Participants with and without diabetes did not differ on their subjective cognitive failures |
| van den Kommer et al. (2014)^40^ | Longitudinal (3-year period) | The Netherlands | | No complaints: 60.0 (2.8)  Complaints: 59.8 (2.8) | N = 471  Drawn from Longitudinal Aging Study Amsterdam (LASA) | Self-report, medications, and general practioner's information: CVD and # chronic conditions | "Do you have problems with memory?" | Cardiovascular disease and number of chronic conditions were not related to memory complaints |
| Wessels et al. (2007)^53^ | Cross-sectional | The Netherlands | | 59.0 (12.2) | N = 100  Hospital outpatients with diabetes | Self-report: CVD | Dutch version of the Cognitive Failures Questionnaire (CFQ) | Participants who reported diabetes reported significantly fewer cognitive failures than the healthy reference group  Self-reported CVD (assessed as one of diabetes-related complications) was not associated with cognitive failures |
| Wong et al. (2012)^44^ | Cross-sectional | Singapore | | NR (50+ years) | N = 515  Drawn from community health assessment | Self-report: diabetes | "Has there been any progressive forgetfulness?" | No difference in having progressive forgetfulness between persons who reported and did not report diabetes |
| Yap et al. (2020)^34^ | Cross-sectional | Malaysia | | NR (56+ years) | N = 6,179  Community-based | Self-report: 7 chronic conditions | “Overall in the last 30 days, how much difficulty did you have with concentrating or remembering things?” | % reported cognitive complaints by group:   - Low comorbidity: 58.7% - Arthritis: 79.3% - Diabetes and hypertension: 65%   Participants with cognitive complaints had the highest odds for heart disease, arthritis, and asthma  Cognitive complaints were associated with multimorbidity |
| Zhang et al. (2017)^30^ | Longitudinal (only baseline results relevant to this review) | United States, Canada, & Puerto Rico | | 67.5 (5.3) | N = 7,540 (males only)  Drawn from the Prevention of Alzheimer’s Disease with Vitamin E and Selenium Study (PREADVISE) | Self-report: 6 chronic conditions | Asked whether they considered their cognitive changes to be consistent with normal aging | No difference in number of comorbidities between those with and without cognitive complaints  % self-reports of each condition in those with/without cognitive complaints:   - Hypertension: 39.7%/39.7% - Diabetes: 11.3%/11.4% - CABG: 4.9%/4.0% - No chronic conditions: 40.3%/46.3% - 1 chronic condition: 39.8%/36.8% - 2 comorbidities: 15.9%/13.7% - 3 comorbidities: 3.5%/2.9% - 4 comorbidities: 0.6%/0.3% |

*Note*. NR = Not reported. COPD – chronic obstructive pulmonary disease. CABG = coronary artery bypass graft. CVD = cardiovascular disease. MI = myocardial infarction. Numeric superscripts indicate the corresponding reference number in the manuscript.
